# Mobile Apps for Speech-Language Therapy in Adults With Communication Disorders: Review of Content and Quality

**DOI:** 10.2196/18858

**Published:** 2020-10-29

**Authors:** Atiyeh Vaezipour, Jessica Campbell, Deborah Theodoros, Trevor Russell

**Affiliations:** 1 RECOVER Injury Research Centre Faculty of Health and Behavioural Sciences The University of Queensland Brisbane Australia; 2 Queensland Aphasia Research Centre Faculty of Health and Behavioural Sciences The University of Queensland Brisbane Australia

**Keywords:** communication disorders, speech therapy, language therapy, ergonomics, rehabilitation, mobile health, mHealth

## Abstract

**Background:**

Worldwide, more than 75% of people with acquired brain injury (ABI) experience communication disorders. Communication disorders are impairments in the ability to communicate effectively, that is, sending, receiving, processing, and comprehending verbal and nonverbal concepts and symbols. Such disorders may have enduring impacts on employment, social participation, and quality of life. Technology-enabled interventions such as mobile apps have the potential to increase the reach of speech-language therapy to treat communication disorders. However, ensuring that apps are evidence-based and of high quality is critical for facilitating safe and effective treatment for adults with communication disorders.

**Objective:**

The aim of this review is to identify mobile apps that are currently widely available to adults with communication disorders for speech-language therapy and to assess their content and quality using the validated Mobile App Rating Scale (MARS).

**Methods:**

Google Play Store, Apple App Store, and webpages were searched to identify mobile apps for speech-language therapy. Apps were included in the review if they were designed for the treatment of adult communication disorders after ABI, were in English, and were either free or for purchase. Certified speech-language pathologists used the MARS to assess the quality of the apps.

**Results:**

From a total of 2680 apps identified from Google Play Store, Apple App Store, and web searches, 2.61% (70/2680) apps met the eligibility criteria for inclusion. Overall, 61% (43/70) were available for download on the iPhone Operating System (iOS) platform, 20% (14/70) on the Android platform, and 19% (13/70) on both iOS and Android platforms. A content analysis of the apps revealed 43 apps for *language*, 17 apps for *speech*, 8 apps for *cognitive communication*, 6 apps for *voice*, and 5 apps for *oromotor function* or *numeracy*. The overall MARS mean score was 3.7 out of 5, SD 0.6, ranging between 2.1 and 4.5, with *functionality* being the highest-scored subscale (4.3, SD 0.6)*, followed by aesthetics* (3.8, SD 0.8), *information* (3.4, SD 0.6)*, and engagement* (3.3, SD 0.6). The top 5 apps were *Naming Therapy* (4.6/5), *Speech Flipbook Standard* (4.6/5), *Number Therapy* (4.5/5), *Answering Therapy*, and *Constant Therapy* (4.4/5).

**Conclusions:**

To our knowledge, this is the first study to systematically identify and evaluate a broad range of mobile apps for speech-language therapy for adults with communication disorders after sustaining ABI. We found a lack of interactive and engaging elements in the apps, a critical factor in sustaining self-managed speech-language therapy. More evidence-based apps with a focus on human factors, user experience, and a patient-led design approach are required to enhance effectiveness and long-term use.

## Introduction

Acquired brain injury (ABI) is a life-changing health condition that can result from trauma, cerebrovascular events, and brain tumors [1]. The population affected by ABI is large and growing, with 69 million individuals sustaining a traumatic brain injury (TBI) globally each year [2], and the global incidence of first stroke is expected to rise from 16 million in 2005 to 23 million in 2030 [3]. More than 75% of people experience a communication disorder after ABI [4]. A communication disorder may involve speech impairment characterized by slurred and indistinct speech (dysarthria or apraxia of speech), specific language impairments characterized by difficulties with comprehension or expression of language (aphasia), communication difficulties associated with cognitive disorders, and impaired social communication skills [5-7]. Post-ABI communication disorders can impact a person’s social integration and participation in their school, work, and community [8]. The quality of life and mood can also be reduced for the affected person and their family members [9]. In addition, communication difficulties may represent significant stressors that influence family and/or caregiver burden [10].

Evidence supports the delivery of rehabilitation by speech-language pathologists (SLPs) for adults after sustaining ABI to treat language [11], motor speech [12], and social communication skill [13] impairments. As the availability of mobile technology increases, apps designed for mobile phones and tablets are increasingly of interest for such therapy. Apps have been developed to identify the presence of aphasia (language impairment) and improve language outcomes [14,15], facilitate homework completion in adults with stroke and TBI [16,17], and improve cognitive skills in adults with acquired cognitive disorders [18]. In a recent cohort study by Munoz et al [16], adults with stroke and TBI used an app targeting speech, language, and cognitive skills. Usage of this app was reported to be higher in geographical areas with limited access to SLP clinics, regardless of demographics such as age. Such apps have the potential to increase the reach of allied health interventions by making therapy available anywhere that a mobile device can be used. The recent global COVID-19 pandemic has highlighted the potential for digital health technologies to provide health care support at a distance [19]. Specifically, mobile therapy apps may increase customization, ease of access, engagement with therapy, and optimize therapy dosage, which could assist in reducing the effects of social stigmas associated with communication impairment [20]. Mobile therapy apps may also offer greater opportunities for generalizing therapy goals to real-world settings and provide additional ways for clients to receive valuable feedback to reinforce positive behaviors and enhance performance [21,22].

Despite the availability and potential benefits of mobile apps for adults with communication disorders after ABI, there is little published evidence regarding their quality beyond the app star rating allocated by some consumers in app store platforms and web-based reviews. A recent analysis of apps for children with speech-language disorders found that most apps were of average quality and that app cost did not always correlate with therapeutic quality [23]. A systematic review of apps targeting general rehabilitation found that some may have a positive impact on outcomes in exercise or gait training or self-management or may be effective as measurement tools [24]. However, only 3 apps targeting communication were included in this general review. The review categorized app functionality without evaluating app qualities such as usability, consumer interaction, or engagement.

In recent years, the term gamification has become increasingly popular in digital health technologies as an underlying element to enhance individuals’ engagement with mobile health technologies. Gamification has been described by Detering et al [25] as the “use of game design elements in nongame contexts,” that is, the use of game design, game playing techniques, and game mechanisms to engage users and motivate positive behavior [25,26]. Previous systematic reviews have reported the positive effects of gamification on health-related interventions [27,28]. Therefore, the design and development of mobile health technologies should focus on relevant, evidence-based therapy goals, and app functionality as well as target positive user experience and sustained engagement (eg, by using gamification principles). To our knowledge, no systematic quality evaluation of apps for adults with communication disorders has been conducted to date. Therefore, an in-depth evaluation of the quality of mobile apps for adults with communication disorders after ABI is needed.

This study aims to (1) identify the available apps designed for adults with communication disorders and (2) evaluate the quality of the available apps using the Mobile App Rating Scale (MARS) [29], a validated tool that has been used to evaluate various medical apps [23,30]. The outcomes of this study will have implications for the design and development of mobile apps as a clinical rehabilitation tool for speech-language therapy.

## Methods

The PRISMA (Preferred Reporting Items for Systematic Reviews and Meta-Analysis) guidelines were adopted for this study. These have been used successfully for evaluating speech-language therapy apps in children with speech disorders [23].

### App Eligibility Criteria

Apps were included in this review if they were primarily designed for adults (18 years or older) with communication disorders secondary to ABI, were for the provision of speech-language therapy (eg, naming drills to improve word-retrieval skills), were in English, were either free or for purchase, were compatible with Android or iPhone Operating System (iOS), and were available on mobile phones and/or tablets.

Apps were excluded if they were primarily designed for children, were not designed for speech-language therapy (eg, designed to teach English as a second language), provided assessment only without speech-language therapy, and were speech-to-text/text-to-speech apps or other augmentative and alternative communication apps.

### App Identification and Search Strategy

To identify mobile apps, a search on Google Play Store (74.6% of the phone and 41.4% tablet market) and Apple App Store (24.8% of the phone and 58.5% of tablet market) was conducted using the following keywords: “*aphasia,” “apraxia,” “dysarthria,” “dyspraxia,” “dysphasia,” “speech*, *articulation,” “speech therapy,” “speech pathology,” “language therapy,” “speech-language pathology,” “speech rehabilitation,”* and “*language rehabilitation”*. The search terms were generated in consultation with 4 certified and experienced SLPs. One researcher entered the terms into the search fields of both the Google Play Store and Apple App Store. Boolean operators were not used as they were not supported by these platforms. In addition, webpages reviewing multiple apps for adults with communication disorders after ABI were reviewed for apps that had not been identified previously. The titles, app platform (ie, iOS or Android), and marketing description of all resulting apps were extracted into a spreadsheet and duplicates were removed. The last search was conducted in November 2019.

### App Screening and Extraction

App screening was performed by an Australian certified SLP using the marketing description of the apps against a list of eligibility criteria. If the SLP could not decide the eligibility, a second reviewer was consulted. The included apps were downloaded to either an Android (Samsung Galaxy) or iOS (iPhone/iPad) device, depending on compatibility, for further evaluation and quality appraisal.

To identify the functionality of the apps for adults with communication disorders following ABI, a coding sheet was developed to categorize the apps based on the description of the primary therapeutic function contained in the app description on the Apple App Store or Google Play Store (ie, communication skills that the app is designed to improve). The coding process was a two-step analysis in which 2 independent researchers reviewed the coding sheet and extracted relevant categories.

### App Quality Appraisal

A total of 3 certified SLPs scored the quality of the included apps using the MARS [29]. The MARS allows raters to assign a 5-point Likert scale rating (1=inadequate to 5=excellent) across 6 categories. The categories were (1) *engagement*, including individual items for entertainment, interest, customization, interactivity, and whether the app was engaging for the target users; (2) *functionality*, including performance, ease of use, navigation, and gestural design; (3) *aesthetics*, including layout, graphics, and visual appeal; and (4) *information quality*, including accuracy of app description; whether the app had specific, measurable, and achievable goals; quality of information; quantity of information; visual information; credibility; and whether the app was evidence-based. Furthermore, 2 additional categories, *subjective quality* and *perceived impact*, captured the rater’s overall impressions. *Subjective quality* included whether the rater would recommend the app (1=definitely not to 5=definitely yes); time predicted by the rater during which the app would be used in the next 12 months, if it was relevant (1=none to 5=>50 times); rater willingness to pay for the app (1=definitely not to 5=definitely yes); and the overall star rating (1=one of the worst apps I have used to 5=one of the best apps I have used). *Perceived impact* was also rated using a 5-point Likert scale (1=strongly disagree to 5=strongly agree) and addressed the app’s perceived potential to increase users’ awareness, knowledge, attitudes, and motivation to complete the desired health behavior, encourage help-seeking, and improve user communication.

As recommended by the MARS developers, each rater viewed the training video by Stoyanov and Hides [29,31]. The raters practiced rating before using the MARS tool and discussed their ratings to agree on the relevance of the MARS items to the SLP apps and to establish a consensus on the MARS items. Each app was trialed for at least 10 min before the rating was completed. When reviewing the apps with the MARS tool, the raters also reviewed app descriptions and developer websites for the availability of published evidence reporting app effectiveness. As there are potential risks associated with mobile health apps, such as inaccurate or out-of-date content [32], the SLP noted any potential safety issues (eg, app provided incorrect feedback to users) or other issues that could potentially hamper therapy progress.

A MARS mean score was calculated for each app in each category, and then a total MARS mean score was calculated from the first 4 categories, with 5 being the highest score possible. The final MARS score was the average score of the 2 raters for each app. The overall MARS score demonstrated a good [33] level of interrater reliability (two-way mixed intraclass correlation coefficient (ICC) 0.61, 95% CI 0.43-0.74) between raters. MARS subscale correlations included engagement, ICC 0.52 (95% CI 0.32-0.68); functionality, ICC 0.60 (95% CI 0.39-0.72); aesthetics, ICC 0.50 (95% CI 0.29-0.66); and information quality, ICC 0.62 (95% CI 0.45-0.75). According to the MARS tool guideline, the total MARS mean scores were used for comparison with consumer app star ratings in the app stores. All correlation data analyses were conducted using SPSS (IBM Corporation).

## Results

### App Overview

**Figure 1:**
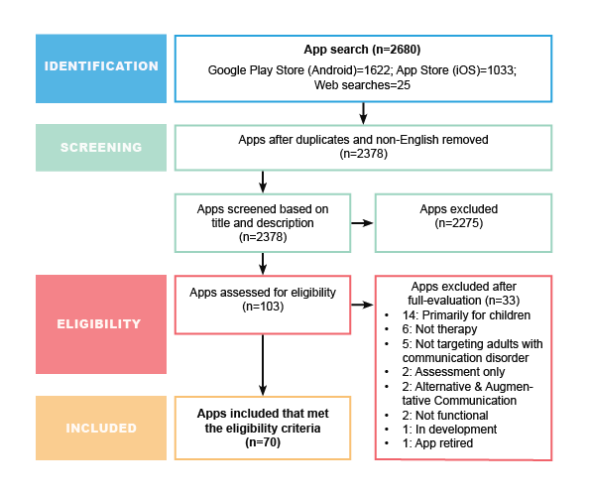
PRISMA (Preferred Reporting Items for Systematic Reviews and Meta-Analysis) flow diagram of app selection.

From a total of 2680 apps identified from Google Play Store, Apple App Store, and web searches, 70 apps met the eligibility criteria. Figure 1 shows the search and selection process. Of the 70 apps included, 43 (61%) were available on the iOS platform, 14 (20%) were available on the Android platform, and 13 (19%) were available on both iOS and Android platforms. A total of 20% (14/70) of apps were completely free to use; 40% (28/70) offered a free or lite version with the option for additional purchase or upgrade; and 34% (24/70) apps needed to be purchased, with prices ranging from Aus $1.99 to $57.99 (US $1.42 to $41.26). Overall, 26% (18/70) apps were available for purchase as a bundle with other apps for a discounted price. Furthermore, 50% (35/70) apps were developed by or in collaboration with SLPs. A total of 29 apps did not have a rating because of an insufficient number of users rating the app. The available star ratings ranged from 1 to 5, with a mean user rating of 3.7 out of 5 (SD 1.2). The Pearson product-moment correlation coefficient analysis was performed to assess the relationship between consumer star ratings and overall MARS ratings. The relationship was not significant (r=.052; n=37; *P*=.76). Multimedia Appendix 1 provides a summary of our findings, and Multimedia Appendix 2 lists the details of all the included apps.

### App Content

The apps identified as primarily targeting adults with communication disorders after ABI were organized by therapeutic purposes (Multimedia Appendix 3). Overall, 43 apps targeted *language therapy*, defined as therapy for comprehending and producing written and spoken words and sentences. Among these language apps, 19 targeted spoken language expression, 15 targeted spoken language comprehension, 13 targeted reading, and 8 targeted writing. A total of 17 apps were designed for *speech therapy*, defined as therapy to improve perception and production of speech sounds and speech segments. This included 10 apps for articulation (individual speech sound production), 6 for motor speech (addressing problems with motor speech planning), and 2 for speech rate. Furthermore, 6 apps were used for *voice therapy*, including 5 addressing vocal loudness and 3 addressing vocal pitch. Overall, 8 apps were designed for *cognitive communication therapy* or cognitive skills underlying communication, such as problem solving, reasoning, inferencing, and executive functions. Finally, 5 apps were designated as *others*, including 3 apps for numbers/numeracy and 1 app for oromotor function (ie, function of oral musculature).

### App Quality

Of the 70 apps, 66 (94%) were rated using the MARS tool. A total of 5 *talk around me apps* were rated together once, as they differed only with regard to the vocabulary items used. A summary of the MARS scores is included in Multimedia Appendix 4.

The average MARS rating was 3.7 out of 5 (SD 0.6; range 2.1-4.5). Overall, 27 apps achieved ratings between 4.0 and 4.9 (27/66, 41%), 30 apps were rated between 3.0 and 3.9 (30/66, 45%), whereas 11% (7/66) apps received ratings between 2.0 and 2.9. No app received a rating of 1 (inadequate). Overall, 86% (57/66) apps were rated as acceptable (3 out of 5) or higher, indicating favorable perceptions of the apps as perceived by certified SLPs.

The top 5 apps were *Naming Therapy*, *Speech Flipbook Standard* (4.6/5), *Number Therapy* (4.5/5), *Answering Therapy*, and *Constant Therapy* (4.4/5). The lowest-rated apps were *Aphasia: Start Talking Again* (2.2/5), *Aphasia Speech Therapy* (2.3/5), *HelpMeTalk*, and *Speech therapy Logopedic Free* (2.5/5). Across all apps identified in the review, *functionality* was the highest-rated subscale (mean 4.3, SD 0.6), followed by *aesthetics* (mean 3.8, SD 0.8), *information* (mean 3.4, SD 0.6), and *engagement* (mean 3.3, SD 0.6).

### Engagement

The top-rated app on the engagement subscale was *Naming Therapy* (4.4/5), as it had a progress bar to introduce an element of gamification, a range of interesting cues, and a number of customization options (eg, ability to alter the number of trials, syllables, add categories/items). *Naming Therapy* also allowed the recording and emailing of the audio recording, provided self-rating options and cues on request, and utilized a simple layout and visual icons suitable for adults with communication disorders. In contrast, the lowest-rated app regarding engagement was *Aphasia Speech Therapy* (1.6/5), which lacked entertaining and engaging elements, did not have a setting page or method for customizing targets, and relied on written targets with no other visual elements to support adults with communication disorders. In addition, the voice recognition function of the *Aphasia Speech Therapy* app did not work at the time of rating (although it may have worked on other devices).

### Functionality

A total of 13 apps received an excellent (5) rating on the functionality subscale for timely app performance, absence of technical issues, being easy and intuitive to use, employing a logical and clear layout, and consistent and intuitive gestural design. The lowest-rated app regarding functionality was *Aphasia: Start Talking Again* (2.1/5), as the speech recognition function was poor and failed to recognize many accurate speech productions. Therefore, the accuracy of the recorded data was poor. In addition, the feedback for speech evaluations was very general in nature and did not provide specific feedback.

### Aesthetic

A total of 2 apps received an excellent (5) rating on the aesthetic subscale. An example of this was *Think Therapy*, which had a simple layout, high-quality graphics, a very attractive color scheme and visual motifs, and seamless animations. *Speech therapy Logopedic Free* was the lowest-rated app on this subscale (2/5). This rating was given because of the unusual layout involving menu items distributed across the screen, activities embedded within activities, its low quality, stylistically inconsistent graphics, and garish color scheme.

### Information

The top 2 apps on the information subscale were *Naming Therapy* (4.5/5) and *Speech Flipbook Standard* (4.4/5). These contained detailed information about how to use the app that incorporated visual information (images of icons and buttons). The information contained in the *Naming Therapy* app is linked to evidence-based techniques, for example, cueing hierarchies and semantic feature analysis [34-36]. Only 4 apps referred to published empirical studies assessing app effectiveness, including one joint trial of *Naming Therapy*, *Reading Therapy*, *Writing Therapy*, and *Comprehension Therapy* and one trial of *Constant Therapy* [17,37]. It is worth mentioning that *Constant Therapy* was the only app to describe the use of a big data approach to collect patient data to personalize therapy and optimize outcomes based on what worked for patients with similar demographics and diagnoses [38]. In contrast, *Aphasia: Start Talking Again*, *HelpMeTalk*, and *Cognitive Rehabilitation 1-3* were rated lowest on this subscale because of poor description of purpose and goals.

### Subjective Quality and Perceived Impact

Only 1 app, *Number Therapy*, received an excellent *subjective quality* rating. Other highly rated apps were *Apraxia Therapy*, *Category Therapy*, *Comprehension Therapy*, *Naming Therapy*, and *Talk Around It* (4.75/5). These apps share a combination of appealing qualities, including multiple ways of interacting with the app (eg, tapping a multiple-choice answer, recording yourself, emailing the recording to others, self-rating, requesting hints), clear intuitive layout, navigation and gestural design, high-quality attractive visuals, and easy ways to track progress and obtain accurate feedback.

Only 2 apps, *Category Therapy* and *Cognifit–Test and Brain Games,* received an excellent *perceived impact* rating. Other highly rated apps included *Naming Therapy*, *Advanced Comprehension Therapy*, *Advanced Naming Therapy*, *Advanced Reading Therapy*, *and Asking Therapy* (4.67/5). With regard to *perceived impact*, in general, apps did not receive ratings for awareness, attitudes, or help-seeking. Rather than building public awareness of communication disorders, changing user attitudes, or encouraging help-seeking, most apps were designed to assist individuals in working on specific communication skills and gaining awareness of their own strengths and weaknesses. Furthermore, apps designed to facilitate repetitive practice of specific communication skills appeared best suited for use as part of a more holistic therapy program directed by an SLP.

Apps that were rated highly on *perceived impact* generally functioned well, accurately rewarded target performance, provided multiple cues/prompts, and offered multiple options for presentation and response modalities. For example, while using *Advanced Comprehension Therapy*, the user can select auditory stimuli, written stimuli, or both; request hints; or request that the stimulus be repeated slowly. In contrast, low-rated apps provided inaccurate feedback (*Aphasia: Start Talking Again*) or rewarded incorrect productions (*Speech therapy Logopedic Free*). Other low-rated apps offered features that were not functional on the device used at the time of the review (eg*, vowel recognition* in *VowelViz Pro* practice function in *Aphasia Word*).

## Discussion

### Principal Findings

This study was designed to identify speech-language therapy mobile apps available in English for adults with communication disorders and evaluate their content and quality using the MARS tool [29]. A total of 70 apps were reviewed on iOS and/or Android platforms in the areas of *speech, language, voice*, *cognitive communication,* *oromotor function*, and/or *numeracy*. A high proportion of the reviewed apps had an average MARS rating of 3.7 out of 5, which is similar to that from previous research by Furlong et al [23] who reviewed 132 apps for children with speech disorders and reported the same average MARS rating of 3.7 out of 5.

This review revealed a lack of clear evidence to demonstrate the clinical benefits of speech-language therapy mobile apps currently available in Google Play Store or Apple App Store for adults with communication disorders. At instances where claims were made for clinical effectiveness, there was a lack of high-quality clinical trials to support these assertions. Overall, 3 of the top 10 apps claimed to be designed and developed based on evidence-based therapy techniques (*Naming Therapy*, *Number Therapy*, and *Apraxia Therapy)*. Despite these claims, only 2 published studies were found that evaluated the clinical effectiveness of these apps. A small pilot, crossover design study evaluated the use of 4 Tactus Therapy apps (*Naming Therapy*, *Comprehension Therapy*, *Reading Therapy*, and *Writing Therapy)* in patients with chronic expressive aphasia. This study found small but significant improvements in language outcomes as measured by a standardized aphasia battery and a narrative discourse measure. However, the number of participants who completed the study was small (n=10), and participants differed significantly in terms of aphasia severity at baseline [37]. In another nonrandomized study [17] involving 51 individuals with aphasia due to stroke or TBI, an iPad-based software platform, *Constant Therapy*, was trialed to ascertain its effects on specific therapy tasks and overall language and cognitive skills. Both the experimental group (n=42) and the control group (n=9) received individual face-to-face clinic sessions once a week for 10 weeks, which involved clinician-assisted delivery of *Constant Therapy* tasks. The experimental group was also asked to use *Constant Therapy* to practice language activities at home, whereas the control group did not do home practice. Small but significant improvements on a standardized aphasia battery were reported in both participant groups*,* with more significant gains evident in the experimental group. However, as the experimental group spent more time on therapy tasks, it is not clear whether the improvement was the result of more opportunities to use *Constant Therapy* or simply the result of more time spent practicing language. In addition, as the participant groups were not matched for severity level at baseline or with regard to time after onset of stroke/TBI, the results may have been confounded. Typically, a degree of improvement over time is expected, especially for individuals still within the first 12 months after onset. Therefore, none of the apps identified in this review had high-level evidence of clinical effectiveness. These findings have important implications for further research with a focus on evaluating the clinical effectiveness of mobile apps. In particular, with the recent worldwide COVID-19 pandemic, it is crucial to offer health care support remotely via digital health technologies [19].

This review showed that the included apps appeared to favor functionality (mean score 4.3, SD 0.6) over aesthetics (mean 3.8, SD 0.8), information (mean 3.4, SD 0.6) and engagement (mean 3.3, SD 0.6). This was surprising given the long-term engagement in speech-language therapy commonly required to gain real health benefits [39]. The idea that patients must be fully engaged in the rehabilitation process to achieve targeted outcomes has been considered analogous to patient participation in compliance with or adherence to this process. Engagement is also facilitated through the relationship and communication between the patient and clinician [39], which can vary considerably depending on individuals’ communication impairment profiles. A possible explanation for the low scores regarding how well apps were able to achieve participant *engagement* might be the lack of human-centered theory-driven approaches [40,41]. Although prior studies on speech therapy mobile apps targeting children have used theory-driven or co-design approaches to improve engagement in therapy [42-44], this is lacking in the design of apps targeting adults with speech-language therapy. In particular, the apps should be tailored to suit individuals’ treatment goals and demographics with a high level of attention to *human factors* [45] to achieve optimal outcomes. It may be challenging for SLPs, unfamiliar with these approaches and methodologies, to develop apps that achieve the desired levels of engagement. The results of this study are consistent with previous research on patient acceptance of consumer health information technology [46], which concluded that there is a need for developers and those who implement the systems to carefully consider the underlying reasons (eg, physical, psychological, and social) for patient acceptance and engagement with technology. A recent review of rehabilitation technology acceptance in adults with TBI also found limited research that comprehensively evaluated usability and user acceptance [47].

A multidisciplinary approach should be taken from the early stages of speech-language therapy app design and development and should involve SLPs, human-computer interaction researchers, user experience researchers, developers, and individuals with communication disorders. This approach would ensure the consideration of both human-centered design [40,48] and evidence-based therapy techniques with attention to the level of engagement, functionality, aesthetics, and information. Further research should be undertaken to investigate how people with communication disorders engage or disengage with mobile-based therapy apps over time in relation to their health goals. There are still unanswered questions about the long-term success of SLP apps accessed by adults with communication disorders.

This review identified limited gamification elements (eg, progress bar, scoring system, self-rating capabilities, ability to request cues/hints) in the included apps. Further research should be undertaken to investigate other game design elements to enhance engagement with rehabilitation technologies by adults with communication disorders. In particular, attention must be paid to inclusive and accessible game design for people with different communication needs and to the inclusion of game elements that are relevant for users of different ages (ie, young vs older adults), experience with technology, cultures, languages, and regional dialects. Future research should also include non-English apps that could be used for bilingual/multilingual patients who should have the opportunity to be treated in their primary or preferred language, irrespective of its popularity. Finally, this review found a lack of relationship between the MARS rating and the in-app consumer star ratings. Similar findings were reported in the study by Knitza et al [30], where MARS had been used to evaluate apps in rheumatology. This is not surprising given that the MARS tool was developed to provide an objective and reliable multidimensional measure of the app quality of health-related apps, that is, engagement, functionality, aesthetics, information, and subjective quality. However, consumer star rating lacks objectivity, and its focus is on usability and popularity among consumers, with little indication of apps’ clinical effectiveness [30].

### Recommendations

The findings of this study have number of recommendations and practical implications.

- More research using a co-design process involving all stakeholders (ie, individuals with communication disorders, SLPs, caregivers) who will use or prescribe the end product is essential for developing apps targeting specific speech-language and communication impairments.
- It is essential to tailor apps to suit targeted patient group profiles by incorporating the capabilities to customize or adjust the content of the apps to simplify or increase complexity to match patient skill levels. For example, incorporation of gamification elements may be a priority to engage young patients with TBI, whereas for older patients affected by stroke, it may be more important to present age-appropriate content in an aphasia-friendly way through the use of visually distinct and appealing pictorial information and simplified language/text and formatting [49].
- Subject matter expert ratings for mobile health apps must be included to provide a more reliable measure of the app’s quality, which could include ratings of engagement, functionality, aesthetics, information, and subjective quality.
- Owing to the increasing number of new app releases in Google Play Store and Apple App Store globally, it is recommended that professional bodies such as Speech-Language Pathology associations establish a database where app developers could register their apps. This could assist with the long-term management of mobile apps and support SLPs in decision making to recommend particular apps to their patients.

### Limitations

A limitation of this review is that details regarding the number of app downloads and demographics of current users were not available, which would have been beneficial for in-depth analysis. Another limitation of this review is that the content and quality of the apps were evaluated solely from the perspective of SLPs and did not include consumers. However, most of these apps were designed to deliver speech-language therapy, where the guidance of SLPs would be instrumental in influencing user uptake. Further research should evaluate the usability of speech-language therapy app and the level of adoption by the target population. In addition, this paper did not review experimental apps currently reported only on journal articles, conference proceedings, or developer websites. Although such apps are not widely available, they may have more theory-driven designs and could show more promise of efficacy for use by adults with communication disorders. Future research focusing on these experimental mobile apps may be worthwhile.

### Conclusions

The results of this study revealed a limited evidence base for speech-language therapy apps and a lack of highly engaging elements such as gamification techniques. Therefore, there is a need for apps to be developed to complement traditional speech-language therapy, utilizing interactive and engaging design elements to enhance user experience and optimize sustainable technology uptake. Furthermore, from the early stages, apps should be designed and developed by a multidisciplinary team of experts, including speech pathologists, human-computer interaction experts, user experience designers, and app developers to ensure a balance between clinically suitable content and positive user experience. Future studies should also ensure the design and development of speech-language therapy apps using patient-led co-design principles by involving adults with communication disorders as codevelopers. If this is done, mobile apps can have the potential to positively enhance the effectiveness and reach of long-term speech-language therapy for adults with communication disorders. Finally, adequately powered randomized controlled trials are needed to assess the efficacy of apps developed for long-term use in the management of adult communication disorders.

## Appendix

Multimedia Appendix 1 Summary of findings.

Multimedia Appendix 2 Summary of the included apps.

Multimedia Appendix 3 List of apps based on their therapeutic purpose.

Multimedia Appendix 4 Mobile App Rating Scale Score by speech pathologists.

## Abbreviations

ABI: acquired brain injury
ICC: intraclass correlation coefficient
iOS: iPhone operating system
MARS: Mobile App Rating Scale
PRISMA: Preferred Reporting Items for Systematic Reviews and Meta-Analysis
SLP: speech-language pathologist
TBI: traumatic brain injury
