# Correction: Mobile Apps for Speech-Language Therapy in Adults With Communication Disorders: Review of Content and Quality

**DOI:** 10.2196/26309

**Published:** 2020-12-11

**Authors:** Atiyeh Vaezipour, Jessica Campbell, Deborah Theodoros, Trevor Russell

**Affiliations:** 1 RECOVER Injury Research Centre Faculty of Health and Behavioural Sciences The University of Queensland Brisbane Australia; 2 Queensland Aphasia Research Centre, Faculty of Health and Behavioural Sciences The University of Queensland Brisbane Australia

In “Mobile Apps for Speech-Language Therapy in Adults With Communication Disorders: Review of Content and Quality” (JMIR Mhealth Uhealth 2020; 8(10):e18858) the authors noted three errors.

In the Results section, App Quality subsection, the top 5 apps were originally listed as developed by Tactus Therapy solutions as follows:

The top 5 apps were Naming Therapy, Speech Flipbook Standard (4.6/5), Number Therapy (4.5/5), Answering Therapy, and Constant Therapy (4.4/5). All of these top apps were made by Tactus Therapy solutions.

This was found to be incorrect as the Constant Therapy app is made by Constant Therapy Health and not by Tactus Therapy solutions. Therefore, the sentence "All of these top apps were made by Tactus Therapy solutions" has been removed from the text to be consistent with the rest of the article which did not describe the name of the app developers.

Similarly, in Multimedia Appendix 1, the sentence “Developed by Tactus Therapy Solutions Ltd” has been deleted.

The contact number of the corresponding author was originally published as "61 7 3346 4824". This has been changed to "61 7 3365 5560".

The correction will appear in the online version of the paper on the JMIR Publications website on December 11, 2020, together with the publication of this correction notice. Because this was made after submission to PubMed, PubMed Central, and other full-text repositories, the corrected article has also been resubmitted to those repositories.

## Appendix

Multimedia Appendix 1 Summary of findings.

