# Supplementary figures and images for: Mobile Apps for Speech-Language Therapy in Adults With Communication Disorders: Review of Content and Quality
Source: JMIR Mhealth Uhealth. 2020 Oct 29;8(10):e18858. doi: 10.2196/18858 (PMC7661246; doi:10.2196/18858)

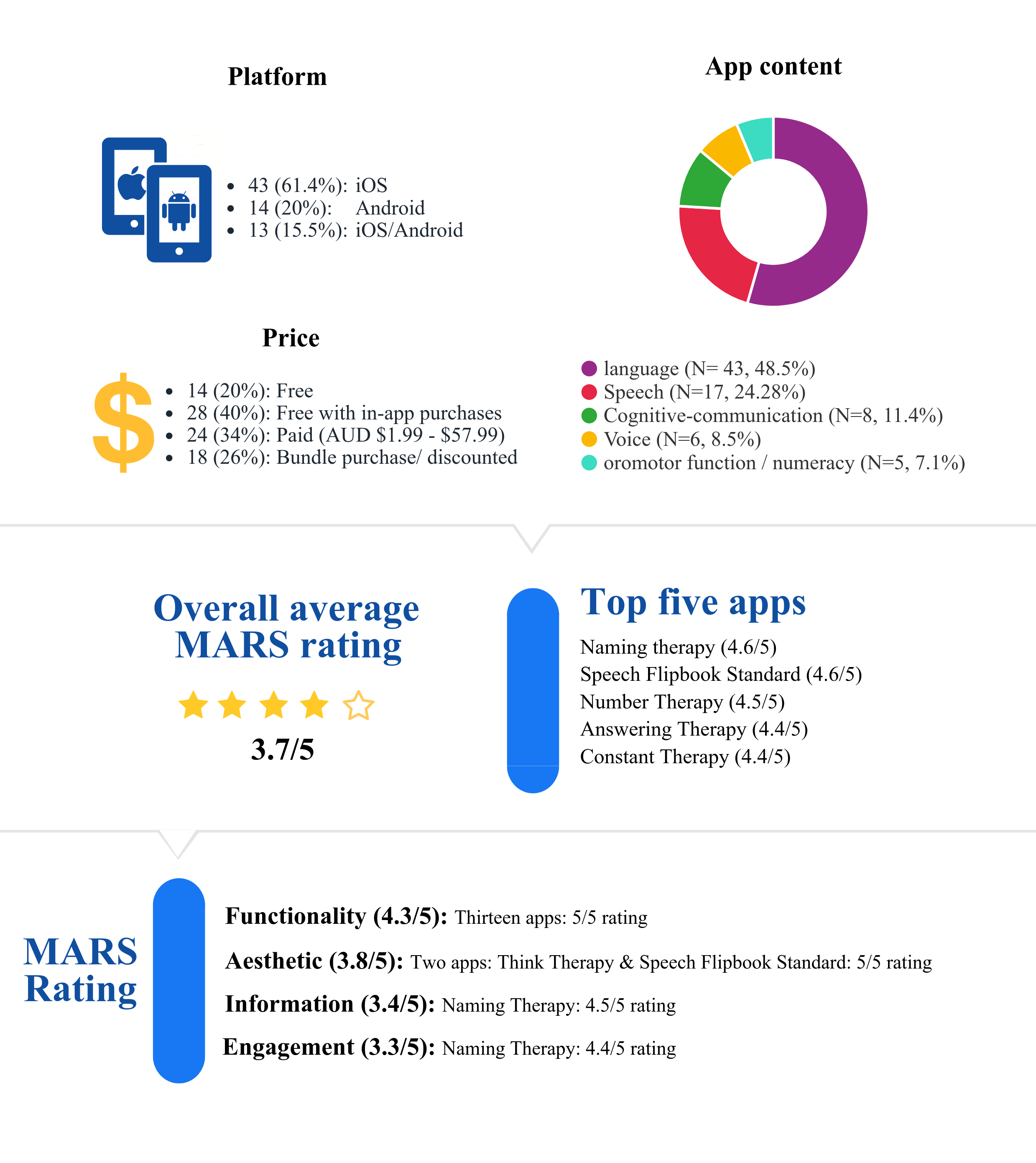

Supplement: Multimedia Appendix 1 [file mhealth_v8i10e18858_app1.png]
