# Supplementary material for: Mobile Apps for Speech-Language Therapy in Adults With Communication Disorders: Review of Content and Quality
Source: JMIR Mhealth Uhealth. 2020 Oct 29;8(10):e18858. doi: 10.2196/18858 (PMC7661246; doi:10.2196/18858)
Supplement: Multimedia Appendix 2 [file mhealth_v8i10e18858_app2.docx]

## Appendix 2. Summary of the included apps.

| **App name** | **Intended user**  **(n.s. = not stated)** | **Category** | **Version** | **Platform (bold = reviewed)** | **When released (v1)** | **Number updates** | **Average update frequency** | **Last update** | **App price** | **Pro/Upgrade price** | **SLP co/ designed** |
| --- | --- | --- | --- | --- | --- | --- | --- | --- | --- | --- | --- |
| Advanced Comprehension Therapy (/Lite) | adults and older children with communication and cognitive impairments | Medical | 1.0.43 | **Android**  /iOS | n/a | n/a | n/a | 31-May-19 | Free (lite) | $38.99 |  |
| Advanced Naming Therapy (/Lite) | people with aphasia | Medical | 1.0.43 | **Android**  **/**iOS | n/a | n/a | n/a | 31-May-19 | Free (lite) | $38.99 |  |
| Advanced Reading Therapy | people with stroke/brain injury | Medical | 1.04 | Android  /**iOS** | Dec-18 | 3 | 3 months | Mar-19 |  | $119.99 |  |
| Advanced Writing Therapy | people with aphasia and other writing disorders | Health & Fitness | 1.0.43 | **Android**  **/**iOS | n/a | n/a | n/a | 31-May-19 |  | $38.99 |  |
| Answering Therapy | children with special needs & adults with acquired communication impairments (aphasia, brain injury) | Medical | 1.07 | Android  /**iOS** | 2014 | 6 | 10 months | Mar-19 |  | $30.99 |  |
| Aphasia Speech Therapy | people with aphasia | Medical | 6 | Android | n/a | n/a | n/a | Sep-3-2018 | Free |  |  |
| Aphasia Words | people with aphasia | Medical | 1 | Android | n/a | n/a | n/a | 27-Aug-17 | Free |  |  |
| Aphasia, Stroke & Dementia aka RecoverBrain | people with aphasia, stroke & dementia | Medical | 5.5.3 | iOS | Jan-19 | 1 | 10 months | Apr-19 |  | $1.49 |  |
| Aphasia: Start Speaking Again | people with aphasia | Medical | n/a | iOS | n/a | n/a | n/a | n/a | $30.99 |  |  |
| Apraxia Therapy (/Lite) | apraxia, aphasia, stroke survivor, family member, speech therapist | Medical | 1.07 | iOS | 2016 | 6 | 6 months | Dec-18 |  | $38.99 |  |
| Articulation flashcards | speech therapists | Medical | 1 | Android | n/a | n/a | n/a | 27-May-17 | $12.99 |  |  |
| Articulation Station Pro | adults & children | Education | 2.6.2 | iOS | 2012 | 21 | 4 months | Nov-18 |  | $89.99 |  |
| Asking therapy | adults with acquired communication impairments (aphasia, brain injury) & children with special needs | Medical | 1.07 | **Android**  **/iOS** | 2014 | 6 | 10 months | Mar-19 |  | $30.99 |  |
| Category Therapy | people with stroke, brain injury, autism, speech therapists | Health & Fitness | 2.01 | **iOS** | 2013 | 8 | 9 months | Jul-19 |  | $22.99 |  |
| Cognifit - Test & Brain Games | healthy individuals and people who suffer some type of cognitive decline or impairment (Dementia, Alzheimer's, Parkinson's, Multiple Sclerosis, Memory or Concentration Problems, Insomnia, Brain Injuries, Learning Disorders, ADHD, Dyslexia, etc.) |  | 3.6.14 | **iOS**  **/Android** | 2018 | 24 | 2 weeks | Sep-19 |  | $149.99/year |  |
| Cognitive Rehabilitation 1 | n.s. | Medical | n/a | iOS | n/a | n/a | n/a | n/a |  | $2.99/level |  |
| Cognitive Rehabilitation 2 | people who have had a stroke or brain injury resulting in aphasia | Health & Fitness | n/a | iOS | n/a | n/a | n/a | n/a |  | $2.99/level |  |
| Cognitive Rehabilitation 3 | people who have had a stroke or brain injury resulting in aphasia | Health & Fitness | n/a | iOS | n/a | n/a | n/a | n/a |  | $2.99/level |  |
| Comprehension Therapy | people with aphasia and cognitive deficits from stroke and other brain injuries | Medical | 3.18 | **iOS**  /Android | 2011 | 18 | 5 months | Feb-19 |  | $38.99 |  |
| Constant Therapy | people with stroke, traumatic brain injury, aphasia, dementia and other speech-language disorders | Medical | 4.8.0 | Android | n/a | n/a | n/a | n/a |  | $25/month |  |
| Conversation Paceboard | people with dysarthria, Parkinson, other clients who need to slow their rate of speech | Medical | 1.3 | iOS | 2013 | 3 | 24 months | 2015 | $10.99 |  |  |
| Conversation Therapy (/Lite) | people who have had a stroke, live with autism, or have a speech and language impairment | Education | 1.3.23 | Android | n/a | n/a | n/a | n/a |  | $38.99 |  |
| Fill in the blank nouns | individuals with significant communication difficulties improve expressive language skills | Education | n/a | iOS | n/a | n/a | n/a | n/a | $9.99 |  |  |
| Following Directions by TSA | n.s. | Education | 1.7 | iOS | 2015 | 6 | 8 months | 2018 | $5.99 |  |  |
| Go-Togethers | individuals of all age groups | Education | 4.1 | iOS | n/a | 8 | 11 months | Jan-19 | $17.99 |  |  |
| HeadApp | people with stroke, traumatic brain injury, brain tumors, psychiatric rehabilitation, geriatrics dementia, aphasia, visual neglect and hemianopia, ADD and ADHD, Multiple Sclerosis, Parkinson's Disease | Health & Fitness | 1.9.6 | Android | n/a | n/a | n/a | 21-Jun-19 | $57.99 |  |  |
| HelpMeTalk | people with speech and language disorders | Medical | 1.6 | Android | n/a | n/a | n/a | 14-Jul-19 |  | $3.79 |  |
| Inference pics | Children with language difficulties, children with autism and adults with brain injury | Medical | n/a | iOS | n/a | n/a | n/a | n/a |  | $22.99 |  |
| Keyword Understanding | children with reduced attention, auditory processing difficulties, receptive language delay, autism, adults with aphasia and cognitive difficulties from stroke or traumatic brain injury | Education | 1.5 | iOS | 2014 | 6 | 12 months | Aug-18 | $22.99 |  |  |
| Language Trainer | people with aphasia, specific language impairment, English Language Learners | Education | 4 | iOS | 2014 | 6 | 10 months | Jan-19 | $17.99 |  |  |
| My Aphasia Coach | people with aphasia | Health & Fitness | 5.9.0 | iOS  /**Android** | n/a | n/a | n/a | 4-Aug-19 |  | $9.99  or $19.99/month |  |
| Naming Toolbox | adults with aphasia who have word retrieval difficulties | Medical | n/a | iOS | n/a | n/a | n/a | n/a | $14.99 |  |  |
| Naming therapy | people with aphasia and children with special needs | Education, Brain Games | 3.1.88 | iOS  /**Android** | n/a | n/a | n/a | 22-Apr-19 | $38.99 |  |  |
| Number therapy | SLPs, people with aphasia or other disorders | Medical | 1.08 | iOS | 2015 | 7 | 7 months | Apr-19 |  | $22.99 |  |
| OLIENA | people with aphasia | Medical | 1.01 | Android | n/a | n/a | n/a | 22-Dec-17 | Free |  |  |
| Reading Rehabilitation Toolkit | adults with reading difficulties | Medical | 3 | iOS | 2013 | 8 | 16 months | Mar-19 | $22.99 |  |  |
| Reading Therapy | adults with neurological impairment (stroke, brain injury, aphasia) and older children with special needs | Medical | 3.1.8 | iOS  /**Android** | n/a | n/a | n/a | 22-Apr-19 |  | $38.99 |  |
| Semantic Links | people with brain injury and aphasia from stroke, older children with special needs including autism | Education | 1.3 | iOS | 2018 | 3 | 4 months | 2018 | $14.99 |  |  |
| Sibilant | people who make /s/ errors | Medical | 4.1 and up | Android | n/a | n/a | n/a | 13-May-18 | Free |  |  |
| SmallTalk Common Phrases | people with apraxia, aphasia, and/or dysarthria resulting from stroke or head injury | Medical | 3.4 | iOS | 2010 | 6 | 20 months | 2015 | Free |  |  |
| SmallTalk Consonant Blends | people with apraxia, aphasia, and/or dysarthria resulting from stroke or head injury | Medical | 3.4 | iOS | 2010 | 6 | 20 months | 2015 | Free |  |  |
| SmallTalk Letters, Numbers, Colours | people with apraxia, aphasia, and/or dysarthria resulting from stroke or head injury | Medical | 3.4 | iOS | 2010 | 6 | 20 months | 2015 | Free |  |  |
| SmallTalk Phonemes | people with apraxia, aphasia, and/or dysarthria resulting from stroke or head injury | Medical | 3.4 | iOS | 2010 | 6 | 20 months | 2015 | Free |  |  |
| Speakup an SPL meter | people that need training modulating or increasing their speaking volume; | Medical | 2.06 | Android | n/a | n/a | n/a | 6-Feb-19 | Free |  |  |
| speech and memory therapy | people with aphasia/Alzheimer's | Medical | 2 | Android | n/a | n/a | n/a | 8-Oct-18 | Free |  |  |
| Speech companion | people with stroke or neurological diseases, such as Parkinson's Disease, multiple sclerosis, or head injury | Education | 3.3 | iOS | n/a | n/a | n/a | 25/02/2019 | $1.99 |  |  |
| Speech Flipbook Standard | SLPS with clients of all ages | Education | 2.04 | iOS | 2013 | 11 | 7 months | Dec-18 | $14.99 |  |  |
| Speech pacesetter | people who have imprecise articulation and fast rate of speech due to stroke, brain injury, stuttering, Parkinson's | Medical | 1.8 | iOS | 2013 | 8 | 9 months | 2018 | $12.99 |  |  |
| Speech Sounds on Cue (Aus) | people with articulation difficulty caused by apraxia of speech or other speech difficulties | Medical | 1.6 | iOS | 2012 | 6 | 14 months | 2018 |  | $19.99 |  |
| Speech therapy logopedic free | adults and children | Medical | 5 | Android | n/a | n/a | n/a | 21-Apr-17 | Free |  |  |
| Speech Trainer 3D | individuals with speech sound disorders and English language learners | Medical | 5 | iOS | 2011 | 8 | 12 months | Feb-18 | $12.99 |  |  |
| Speech Tutor (/Pro) | SLP, SLP student, parent of child with speech delays, someone trying to improve their American English accent | Education | 3.2.4 | iOS | 2011 | 16 | 6 months | Dec-18 | $30.99/  62.99 |  |  |
| SpeechBox for Speech Therapy | children and adults with childhood apraxia of speech, dyspraxia, autism, Down's syndrome, stroke rehabilitation, articulation, and phonological disorders | Education | 2.3.4 | iOS | 2013 | 21 | 3 months | Jan-19 | $5.99/  11.99  /15.49/  month |  |  |
| Talk around it Home (lite)/ Talk Around it USA Free/Talk Around it Speech Therapy/ Talk Around It Nature/Talk Around it Men/Talk Around it Personal | people with aphasia, anomia, stroke, dementia, Alzheimer's and autism | Medical | 2.01 | iOS | 2013 | 7 | 10 months | 2017 |  | $27.99 |  |
| Talkpath News | individuals who need help reading, listening to or understanding the daily news. | News & Magazines | n/a | iOS | n/a | n/a | n/a | n/a | Free |  |  |
| Think Therapy | people with cognitive impairment | Education | 2.1.0 | Android | n/a | n/a | n/a | 1-Aug-19 |  | $9.99/month |  |
| Verb Toolbox | children and adults who need to improve their spoken and written verb comprehension, verb retrieval and naming, verb production in sentences | Education | 2.1 | iOS | 2015 | 8 | 6 months | Dec-18 | $14.99 |  |  |
| Verbal Naming for Aphasia | people with aphasia who have anomia or word finding problems | Education | n/a | iOS | n/a | n/a | n/a | n/a | Free |  |  |
| Voice Analyst | Speech and language therapists / pathologists | Medical | 3.6.9 | Android | n/a | n/a | n/a | 18-Feb-19 | $14.99 |  |  |
| Voice Meter Pro | any child or adult who needs to monitor volume of their own voice | Education | 1.7.1 | iOS | 2013 | 9 | 8 months | Aug-19 | $7.99 |  |  |
| Voice Tools: Pitch, Tone, & Volume | transgender, LSVT, and voice therapy patients | Medical | 1.00.34 | **Android**  /iOS | n/a | n/a | n/a | n/a | Free |  |  |
| Voice Volume Meter Pro | SLPS, people who need help controlling volume | Medical | n/a | iOS | n/a | n/a | n/a | n/a | $5.99 |  |  |
| VowelViz (/Pro) | anyone who needs to practice vowels | Education | 1.1.6 | iOS | n/a | 6 | 10 months | 2015 | $30.99/  79.99 |  |  |
| VoxTraining-Equilibrist | n.s. | Education | 1.1.0 | iOS | 2015 | 3 | 16 months | May-19 | $27.99 |  |  |
| Word Vault Essential (/Pro) | children and adults, SLPs, parents, teachers, professionals | Education | 5.1 | iOS | 2015 | 13 | 4 months | Mar-19 |  | $89.99/year |  |
| Writing Therapy | adults with brain injury or stroke, ESL speakers, children learning to spell | Medical | 3.1.88 | iOS  /**Android** | n/a | n/a | n/a | Apr-22-2019 |  | $38.99 |  |
