# Supplementary material for: Mobile Apps for Speech-Language Therapy in Adults With Communication Disorders: Review of Content and Quality
Source: JMIR Mhealth Uhealth. 2020 Oct 29;8(10):e18858. doi: 10.2196/18858 (PMC7661246; doi:10.2196/18858)
Supplement: Multimedia Appendix 3 [file mhealth_v8i10e18858_app3.docx]

**Appendix 3.** List of apps for individuals with communication disorders based on their therapeutic purpose.

| **App** | **Language** | **Speech** | **Cognitive-communication** | **Voice** | **Other** | **MARS**  **Mean score** |
| --- | --- | --- | --- | --- | --- | --- |
| **Definition** | Comprehending and producing written and spoken words and sentences | Perceiving and producing speech sounds and speech segments | Using cognitive skills such as problem-solving, reasoning, inferencing, executive functions in verbal/non-verbal communication | Vocal quality, pitch and loudness | Oro-motor function, numbers/numeracy |  |
| Advanced Comprehension Therapy (/Lite) | ✓ |  |  |  |  | 4.3 |
| Advanced Naming Therapy (/Lite) | ✓ |  |  |  |  | 4.3 |
| Advanced Reading Therapy (/Lite) | ✓ |  |  |  |  | 4.2 |
| Advanced Writing Therapy (/Lite) | ✓ |  |  |  |  | 4.3 |
| Answering Therapy (/Lite) | ✓ |  |  |  |  | 4.4 |
| Aphasia Speech Therapy | ✓ |  |  |  |  | 2.3 |
| Aphasia Words | ✓ |  |  |  |  | 3.3 |
| Aphasia, Stroke & Dementia aka RecoverBrain | ✓ |  | ✓ |  | ✓ | 4.1 |
| Aphasia: Start Talking Again | ✓ |  |  |  |  | 2.2 |
| Apraxia Therapy (/Lite) |  | ✓ |  |  |  | 4.3 |
| Articulation Flashcards |  | ✓ |  |  |  | 3.9 |
| Articulation Station Pro |  | ✓ |  |  |  | 3.9 |
| Asking Therapy (/Lite) | ✓ |  |  |  |  | 4.3 |
| Category Therapy (/Lite) | ✓ |  |  |  |  | 4.2 |
| CogniFit – Test & Brain Games |  |  | ✓ |  |  | 4.3 |
| Cognitive Rehabilitation 1 |  |  | ✓ |  |  | 3.3 |
| Cognitive Rehabilitation 2 |  |  | ✓ |  |  | 2.7 |
| Cognitive Rehabilitation 3 |  |  | ✓ |  |  | 2.7 |
| Comprehension Therapy | ✓ |  |  |  |  | 3.5 |
| Constant Therapy | ✓ |  | ✓ |  |  | 4.4 |
| Conversation Paceboard |  | ✓ |  |  |  | 3.8 |
| Conversation Therapy (/Lite) | ✓ |  |  |  |  | 3.7 |
| Fill in the Blank Nouns | ✓ |  |  |  |  | 3.5 |
| Following Directions by TSA | ✓ |  |  |  |  | 3.9 |
| Go-Togethers | ✓ |  |  |  |  | 3.8 |
| HeadApp | ✓ |  | ✓ |  |  | 3.9 |
| Help Me Talk | ✓ |  |  |  |  | 2.5 |
| Inference pics (/Lite) |  |  | ✓ |  |  | 4.3 |
| Keyword Understanding | ✓ |  |  |  |  | 4.2 |
| Language Trainer | ✓ |  |  |  |  | 4.0 |
| My Aphasia Coach | ✓ |  |  |  | ✓ | 3.8 |
| Naming Therapy | ✓ |  |  |  |  | 4.6 |
| Naming Toolbox | ✓ |  |  |  |  | 4.3 |
| Number Therapy (/Lite | ✓ |  |  |  | ✓ | 4.5 |
| OLIENA | ✓ |  |  |  |  | 2.7 |
| Reading Rehabilitation Toolkit | ✓ |  |  |  |  | 3.8 |
| Reading Therapy | ✓ |  |  |  |  | 4.3 |
| Semantic Links | ✓ |  |  |  |  | 4.3 |
| Sibilant |  | ✓ |  |  |  | 3.5 |
| SmallTalk Common Phrases |  | ✓ |  |  |  | 2.8 |
| SmallTalk Consonant Blends |  | ✓ |  |  |  | 3.3 |
| SmallTalk Letters, Numbers, Colours |  | ✓ |  |  |  | 3.3 |
| SmallTalk Phonemes |  | ✓ |  |  |  | 3.0 |
| Speak Up: An SPL Meter |  |  |  | ✓ |  | 4.0 |
| Speech and Memory Therapy | ✓ |  |  |  | ✓ | 3.0 |
| Speech Companion |  |  |  |  | ✓ | 3.3 |
| Speech FlipBook Standard | ✓ | ✓ |  |  |  | 4.6 |
| Speech Pacesetter |  | ✓ |  |  |  | 4.1 |
| Speech Sounds on Cue (Aus Eng) (/US Eng/Lite Aus) |  | ✓ |  |  |  | 3.7 |
| Speech Therapy Logopedic Free | ✓ |  |  |  |  | 2.5 |
| Speech Trainer 3D |  | ✓ |  |  |  | 3.7 |
| Speech Tutor (/Pro) |  | ✓ |  |  |  | 3.8 |
| SpeechBox for Speech Therapy |  | ✓ |  |  |  | 4.3 |
| Talk Around it Home | ✓ |  |  |  |  | 3.6 |
| Talk Around it (Men) | ✓ |  |  |  |  | 3.6 |
| Talk Around it (Nature) | ✓ |  |  |  |  | 3.6 |
| Talk Around it (Personal) | ✓ |  |  |  |  | 3.6 |
| Talk Around it (USA) | ✓ |  |  |  |  | 3.6 |
| TalkPath News | ✓ |  |  |  |  | 3.8 |
| Think Therapy | ✓ |  |  |  |  | 4.1 |
| Verb Toolbox | ✓ |  |  |  |  | 4.2 |
| Verbal Naming for Aphasia | ✓ |  |  |  |  | 3.3 |
| Voice Analyst |  |  |  | ✓ |  | 4.0 |
| Voice Meter Pro |  |  |  | ✓ |  | 3.7 |
| Voice tools: Pitch, Tone & Volume |  |  |  | ✓ |  | 3.4 |
| Voice Volume Meter Pro |  |  |  | ✓ |  | 3.5 |
| VowelViz (Pro) |  | ✓ |  |  |  | 3.8 |
| VoxTraining - Equilibrist |  |  |  | ✓ |  | 3.3 |
| Word Vault Essential (/Pro) | ✓ | ✓ |  |  |  | 3.6 |
| Writing Therapy | ✓ |  |  |  |  | 4.2 |
