# Supplementary material for: Mobile Apps for Speech-Language Therapy in Adults With Communication Disorders: Review of Content and Quality
Source: JMIR Mhealth Uhealth. 2020 Oct 29;8(10):e18858. doi: 10.2196/18858 (PMC7661246; doi:10.2196/18858)
Supplement: Multimedia Appendix 4 [file mhealth_v8i10e18858_app4.docx]

**Appendix 4.** MARS Score by Speech Pathologists.

| **App name** | **Engagement** | **Functionality** | **Aesthetics** | **Information** | **MARS Mean** | **Subjective**  **quality** | **Perceived Impact** | **Consumer App Store rating** | **Number of App Store raters** |
| --- | --- | --- | --- | --- | --- | --- | --- | --- | --- |
| Advanced Comprehension Therapy (/Lite) | 3.8 | 5.0 | 4.7 | 3.8 | 4.3 | 4.50 | 4.67 | n/a | No ratings |
| Advanced Naming Therapy (/Lite) | 3.7 | 5.0 | 4.7 | 3.8 | 4.3 | 4.50 | 4.67 | n/a | No ratings |
| Advanced Reading Therapy | 3.8 | 4.9 | 4.3 | 3.7 | 4.2 | 4.50 | 4.67 | 5 | 1 |
| Advanced Writing Therapy | 3.9 | 5.0 | 4.5 | 3.7 | 4.3 | 4.50 | 4.33 | n/a | No ratings |
| Answering Therapy | 4.1 | 5.0 | 4.7 | 3.7 | 4.4 | 4.00 | 4.33 | n/a | No ratings |
| Aphasia Speech Therapy | 1.6 | 3.1 | 2.3 | 2.2 | 2.3 | 1.75 | 1.50 | 4 | 53 |
| Aphasia Words | 2.8 | 3.4 | 4.3 | 2.7 | 3.3 | 1.25 | 1.67 | 2.8 | 6 |
| Aphasia, Stroke & Dementia aka RecoverBrain | 3.6 | 4.8 | 4.5 | 3.4 | 4.1 | 3.50 | 4.33 | n/a | No ratings |
| Aphasia: Start Talking Again | 2.5 | 2.1 | 2.5 | 1.7 | 2.2 | 1.00 | 1.00 | n/a | No ratings |
| Apraxia Therapy (/Lite) | 4.1 | 4.8 | 4.7 | 3.7 | 4.3 | 4.75 | 3.67 | n/a | No ratings |
| Articulation flashcards | 3.1 | 5.0 | 3.7 | 3.7 | 3.9 | 2.50 | 2.00 | n/a | No ratings |
| Articulation Station Pro | 2.7 | 4.9 | 3.8 | 4.0 | 3.9 | 3.50 | 4.33 | 3.8 | 36 |
| Asking therapy | 4.2 | 4.9 | 4.7 | 3.4 | 4.3 | 4.25 | 4.67 | n/a | No ratings |
| Category Therapy | 4.1 | 4.9 | 4.2 | 3.8 | 4.2 | 4.75 | 5.00 | 5 | 1 |
| Cognifit - Test & Brain Games | 4.0 | 5.0 | 4.7 | 3.4 | 4.3 | 3.50 | 5.00 | 3.8 | 13 |
| Cognitive Rehabilitation 1 | 2.9 | 4.1 | 3.3 | 2.7 | 3.3 | 2.75 | 2.33 | n/a | No ratings |
| Cognitive Rehabilitation 2 | 2.0 | 4.5 | 2.2 | 2.0 | 2.7 | 2.75 | 2.33 | n/a | No ratings |
| Cognitive Rehabilitation 3 | 2.1 | 4.5 | 2.3 | 2.0 | 2.7 | 2.75 | 2.33 | n/a | No ratings |
| Comprehension Therapy | 2.9 | 4.8 | 3.5 | 2.9 | 3.5 | 4.75 | 4.33 | 5 | 1 |
| Constant Therapy | 4.2 | 4.9 | 4.3 | 4.1 | 4.4 | 4.25 | 4.00 | 4.1 | 453 |
| Conversation Paceboard | 3.4 | 4.5 | 3.7 | 3.7 | 3.8 | 3.50 | 3.00 | n/a | No ratings |
| Conversation Therapy (/Lite) | 3.1 | 4.4 | 3.8 | 3.5 | 3.7 | 2.50 | 3.67 | 3.7 | 13 |
| Fill in the blank nouns | 3.3 | 4.0 | 3.5 | 3.0 | 3.5 | 3.25 | 3.33 | n/a | No ratings |
| Following Directions by TSA | 3.1 | 4.1 | 4.7 | 3.7 | 3.9 | 2.00 | 2.00 | 2 | 1 |
| Go-Togethers | 3.5 | 3.9 | 4.2 | 3.8 | 3.8 | 4.00 | 4.33 | 5 | 1 |
| HeadApp | 3.9 | 4.4 | 3.5 | 3.9 | 3.9 | 3.50 | 4.33 | n/a | No ratings |
| HelpMeTalk | 2.6 | 3.1 | 2.2 | 2.0 | 2.5 | 2.25 | 1.67 | 4.3 | 7 |
| Inference pics | 4.0 | 4.9 | 4.7 | 3.6 | 4.3 | 4.25 | 4.00 | n/a | No ratings |
| Keyword Understanding | 3.8 | 4.6 | 4.5 | 3.8 | 4.2 | 4.00 | 3.67 | n/a | No ratings |
| Language Trainer | 3.7 | 4.6 | 3.5 | 4.0 | 4.0 | 4.00 | 4.33 | n/a | No ratings |
| My Aphasia Coach | 3.5 | 3.8 | 4.0 | 3.8 | 3.8 | 4.25 | 4.33 | 2.5 | 6 |
| Naming therapy | 4.4 | 5.0 | 4.3 | 4.5 | 4.6 | 4.75 | 4.67 | n/a | No ratings |
| Naming Toolbox | 3.7 | 5.0 | 4.7 | 3.9 | 4.3 | 4.25 | 4.00 | 4.5 | 13 |
| Number therapy | 4.1 | 5.0 | 4.7 | 4.1 | 4.5 | 5.00 | 4.33 | n/a | No ratings |
| OLIENA | 2.1 | 3.3 | 2.3 | 3.0 | 2.7 | 1.00 | 1.00 | 4.4 | 10 |
| Reading Rehabilitation Toolkit | 3.8 | 4.1 | 4.3 | 3.1 | 3.8 | 3.25 | 3.67 | 1 | 1 |
| Reading Therapy | 4.1 | 4.8 | 4.2 | 4.1 | 4.3 | 4.25 | 3.67 | 3.9 | 49 |
| Semantic Links | 4.0 | 4.9 | 4.5 | 3.8 | 4.3 | 4.00 | 3.67 | n/a | No ratings |
| Sibilant | 3.3 | 4.0 | 3.5 | 3.2 | 3.5 | 2.50 | 3.00 | 5 | 1 |
| SmallTalk Common Phrases | 2.2 | 3.6 | 2.3 | 3.1 | 2.8 | 3.50 | 3.00 | n/a | No ratings |
| SmallTalk Consonant Blends | 2.3 | 4.5 | 3.7 | 2.9 | 3.3 | 3.50 | 2.67 | 2 | 1 |
| SmallTalk Letters, Numbers, Colours | 2.3 | 4.5 | 3.7 | 2.9 | 3.3 | 3.50 | 3.00 | 1 | 1 |
| SmallTalk Phonemes | 2.2 | 3.9 | 2.8 | 3.0 | 3.0 | 3.50 | 3.00 | 1 | 1 |
| Speakup an SPL meter | 3.2 | 4.8 | 4.0 | 3.9 | 4.0 | 2.50 | 3.33 | 4.7 | 9 |
| speech and memory therapy | 3.2 | 3.1 | 2.5 | 3.1 | 3.0 | 2.50 | 3.00 | 5 | 1 |
| Speech companion | 3.0 | 4.0 | 3.2 | 3.1 | 3.3 | 2.50 | 3.33 | 4.2 | 24 |
| Speech Flipbook Standard | 4.1 | 4.8 | 5.0 | 4.4 | 4.6 | 4.50 | 3.67 | 5 | 1 |
| Speech pacesetter | 3.7 | 4.6 | 4.2 | 3.9 | 4.1 | 4.00 | 3.67 | 3.8 | 4 |
| Speech Sounds on Cue (Aus) | 3.3 | 4.6 | 3.7 | 3.3 | 3.7 | 4.25 | 4.00 | 4.5 | 2 |
| Speech therapy logopedic free | 2.2 | 3.5 | 2.0 | 2.3 | 2.5 | 1.00 | 1.00 | 3.2 | 21 |
| Speech Trainer 3D | 2.9 | 3.9 | 4.5 | 3.4 | 3.7 | 4.00 | 4.00 | 3 | 2 |
| Speech Tutor (/Pro) | 3.2 | 4.1 | 4.3 | 3.4 | 3.8 | 4.00 | 4.00 | 1 | 1 |
| SpeechBox for Speech Therapy | 4.1 | 4.5 | 4.7 | 3.8 | 4.3 | 4.00 | 3.00 | 3.5 | 13 |
| Talk around it Home (lite)/ Talk Around it USA Free/Talk Around it Speech Therapy/ Talk Around It Nature/Talk Around it Men/Talk Around it Personal | 3.1 | 4.1 | 3.5 | 3.5 | 3.6 | 4.75 | 3.33 | n/a | No ratings |
| Talkpath News | 3.2 | 5.0 | 3.7 | 3.4 | 3.8 | 4.00 | 3.00 | n/a | No ratings |
| Think Therapy | 3.4 | 5.0 | 5.0 | 3.0 | 4.1 | 3.00 | 3.67 | 3.6 | 10 |
| Verb Toolbox | 3.6 | 5.0 | 4.33 | 3.7 | 4.2 | 4.50 | 4.00 | n/a | No ratings |
| Verbal Naming for Aphasia | 3.3 | 3.4 | 2.33 | 3.1 | 3.3 | 2.75 | 2.67 | n/a | No ratings |
| Voice Analyst | 3.4 | 5.0 | 4.00 | 3.6 | 4.0 | 3.75 | 4.00 | 3.8 | 25 |
| Voice Meter Pro | 2.9 | 4.9 | 3.00 | 3.5 | 3.7 | 2.25 | 3.00 | 5 | 2 |
| Voice Tools: Pitch, Tone, & Volume | 2.9 | 4.4 | 2.67 | 3.5 | 3.4 | 3.25 | 4.00 | 3.9 | 65 |
| Voice Volume Meter Pro | 3.4 | 3.9 | 3.33 | 3.8 | 3.5 | 4.00 | 3.67 | n/a | No ratings |
| VowelViz (/Pro) | 2.7 | 3.5 | 4.67 | 4.3 | 3.8 | 1.25 | 1.00 | n/a | No ratings |
| VoxTraining-Equilibrist | 3.5 | 3.5 | 2.67 | 3.7 | 3.3 | 4.00 | 4.33 | n/a | No ratings |
| Word Vault Essential (/Pro) | 3.5 | 3.9 | 3.5 | 3.6 | 3.6 | 3.75 | 3.33 | 4.4 | 21 |
| Writing Therapy | 3.6 | 4.9 | 3.67 | 3.9 | 4.2 | 4.25 | 4.33 | 3.9 | 49 |
